# Transcriptomics-Based Study of Differentially Expressed Genes Related to Fat Deposition in Tibetan and Yorkshire Pigs

**DOI:** 10.3389/fvets.2022.919904

**Published:** 2022-06-09

**Authors:** Xinglong Gong, Min Zheng, Jian Zhang, Yourong Ye, Mengqi Duan, Yangzom Chamba, Zhongbin Wang, Peng Shang

**Affiliations:** ^1^Tibet Agriculture and Animal Husbandry College, Tibet, China; ^2^The Provincial and Ministerial Co-founded Collaborative Innovation Center for R&D in Tibet Characteristic Agricultural and Animal Husbandry Resources, Tibet, China

**Keywords:** Tibetan pig, large white pig, fat deposition, transcriptomic, backfat

## Abstract

Fat deposition traits are one of the key factors in pig production and breeding. The fat deposition capacity of pigs mainly affects the quality of pork and pig productivity. The aim of this study was to analyze the differential expression of mRNA levels in dorsal adipose tissue of Tibetan and York pigs at different growth stages using transcriptomic data to estimate key genes that regulate fat deposition in pigs. The results showed that a total of 32,747 positively expressed genes were present in the dorsal adipose tissue of the two breeds. Differentially expressed gene (DEG) screening of multiple combinations between the two breeds yielded 324 DEGS. Gene ontology (GO) biofunctional enrichment and Kyoto Encyclopedia of Genes and Genomes (KEGG) analyses showed that these DEGS were mainly involved in lipid metabolic pathways, steroid biosynthetic pathways and lipid biosynthetic processes, sterol biosynthetic processes, brown adipocyte differentiation, and other pathways related to lipid deposition and metabolism. The results showed that ACACA, SLC2A4 and THRSP genes positively regulated the lipid deposition ability and CHPT1 gene negatively regulated the lipid deposition ability in pigs. The results of this experiment suggest a theoretical basis for further studies on the regulatory mechanisms of fat deposition in pigs.

## Introduction

In China, pigs are commonly used for meat production and hundreds millions people need meat products from pigs (1, 2). Fat is a metabolic tissue that plays an important role in fatty acid synthesis, and mediates adipocyte differentiation and metabolism (3, 4). At the time of selection of pig for breeding, meat quality traits are considered important indicators while the intramuscular fat (IMF) contents directly affect the sensory quality of pork (5–7). The process of fat deposition is regulated by a cascade of multiple transcription factors including lipid secretion factors, and porcine fat deposition is a complex multiple micro-effective gene control quantitative trait (8, 9). York pigs breed with high leanness is largely introduced in China which is very much in line with modern people's requirements for pork diet and is one of the main sources of pork food in China (10). Tibetan pigs are smaller and a rough-feeding tolerant pig breed with high fat deposition which is a preferred test animal for studying fat deposition traits (11, 12). Therefore, these two breeds can be used as comparative models to study the genetic differences and molecular mechanisms of the phenotypic differences.

At present, there are many studies on fat deposition-related genes, and new genes are gradually discovered, but there is still a need to explore the involvement of potential candidate genes in deposition of fat. RNA-seq technology is a transcriptome analysis tool that provides great advantages for exploring new genes and their related functional properties (13–15). In order to investigate the relationship between different genes in fat deposition in different growth stages of different breeds of pigs, this experiment took Tibetan pigs, a fatty breed, and Yorkshire pigs, a lean breed, as the subjects of the study, and explored three key time points, namely the weaning period of 30-day-old piglets, the fattening period of 90-day-old pigs and the breeding period of 180-day-old pigs. The involvement of each gene in the regulation of lipid deposition and lipid metabolism in dorsal fat tissue was determined by transcriptome sequencing analysis in order to provide a scientific basis for quality selection and breeding of Tibetan and Yorkshire pigs.

## Materials and Methods

### Sample Preparation

The test animals like Tibetan pigs (TP) and York pigs (LW) were reared in the teaching practice pasture of Tibet Agricultural and Animal Husbandry College, Linzhi, Tibet. The dorsal fat tissue was used as the study material. The test animals were reared according to the national feed standard until 30, 90, and 180 days of age, and 8 animals with similar growth conditions and age were randomly selected for slaughtering. A total of 24 Tibetan pigs and 24 York pigs were slaughtered. Dorsal lipid tissues were collected and immediately placed in liquid nitrogen. After collection, all the samples were stored at −80°C for extraction of total RNA. Finally, total RNA of 8 pigs at the same age stage was randomly mixed into 4 biological replicates for transcriptome sequencing. All the research work was carried out in strict accordance with the guidelines approved by the Animal Welfare Committee of Tibet Agriculture and Animal Husbandry University.

### RNA Isolation and Library Construction

Total RNA was extracted from back adipose tissue using TRIZOL reagent (Invitrogen, San Diego, CA, USA) according to the manufacturer's instructions. Degradation and contamination of RNA were monitored on a 1% agarose gel. RNA purity was checked using a NanoPhotometer^®^ spectrophotometer (IMPLEN, CA, USA). RNA integrity was assessed using the Bioanalyzer 2100 system (Agilent Technologies, CA, USA) using RNA Nano 6000 Assay Kit. A total of 1 μg of RNA per sample was used as input material for RNA sample preparation. Sequencing libraries were generated using the NEBNext^®^ UltraTM RNA Library Prep Kit for Illumina^®^ (NEB, USA) following the manufacturer's recommendations, and index codes were added to group sequences into each sample. After library construction, initial quantification was performed using a Qubit2.0 Fluorometer, diluting the library to 1.5 ng/ul, followed by detection of the insert size of the library using an Agilent 2100 bioanalyzer, and after the insert size met expectations, qRT-PCR was performed to accurately quantify the effective concentration of the library. After the insert size met the expectation, qRT-PCR was performed to accurately quantify the effective concentration of the library (effective library concentration above 2nM) to ensure the quality of the library.

### Data Analysis of RNA-Seq

Data analysis included quality control of raw reads, filtering, alignment, assembly, expression count, and annotation. Raw data (raw reads) of fastq format were firstly processed through in-house perl scripts. In this step, clean data (clean reads) were obtained by removing reads containing adapter, reads containing ploy-N and low quality reads from raw data. At the same time, Q20, Q30 and GC content the clean data were calculated. All the downstream analyses were based on the clean data with high quality. Clean reads were mapped to the reference genome (Sus scrofa genome v11.1, downloaded from ENSEMBL web server) using HISAT2 v2.1.0. Reads for each gene in each sample were counted by HTSeq v0.6.0 (https://github.com/simonanders/htseq). Fragments per kilobase per million mapped reads were then calculated to estimate the expression level of each gene. The differentially expressed genes (DEGs) were identified using the statistical significance of the absolute value of |log2FoldChange| >0 and padj <0.01.

### Functional Enrichment Analysis

Matescape (http://metascape.org/) was used to analyze differential genes. Go and KEGG analysis was performed using the OmicShare tools, a free online platform for data analysis (https://www.omicshare.com/tools.From). Cellular Component (CC), Molecular Function (MF) and Biological Process (BP) functions are described. This experiment uses Go and KEGG are both commonly used bioinformatics analysis methods.

### Verification of RNA-Seq Data

To validate the results of sequencing analysis, 7 mRNAs were selected for RT-qPCR analysis (Supplementary Table S1). Total RNA from each sample was extracted using TRIzol reagent (Invitrogen, Carlsbad, CA, U.S.A.), and All-in-one TM First-Strand cDNA Synthesis Kit was used for reverse transcription PCR in accordance with the manufacturer's instructions (GeneCopocie, Inc, Rockville, MD, U.S.A.). qPCR was performed using 2 × All-in-oneTMqPCR mix with 2 μl of cDNA template and a final concentration of 0.2 μl All-in-oneTMqPCR primer. The reactions were carried out as follows: 10 min at 95°C (1 cycle), 20 sec at 95°C, 10 sec at 60°C and 10 sec at 72°C (38 cycles).

### Correlation Analysis of RT-qPCR and Pig Backfat Thickness

The results of RT-qPCR tests of the seven key candidate genes screened in this experiment were used as material for correlation analysis with backfat thickness of Tibetan and York pigs at 180 days of age to further demonstrate the regulatory functions of these genes on fat deposition traits in pigs.

## Results

### RNA Sequencing Data Mapping and Annotation

The quality of sequencing data for all samples in this experiment is summarized in Supplementary Table S2. After filtering the raw data, the amount of data for each sample ranged between 44.49 to 5.43 million clean reads, and the average Clean bases per sample was not <6.6 Gb, and the comparison rate was above 93 %, among which the number of reads compared to multiple positions of the reference genome did not exceed 2.5 %. Q20 was above 97.23% and Q30 was above 92.38%. This indicates that the sequencing quality is high and the valid sequences can be used for subsequent analysis.

### Cluster Analysis of Differential Genes

**Figure 1:**
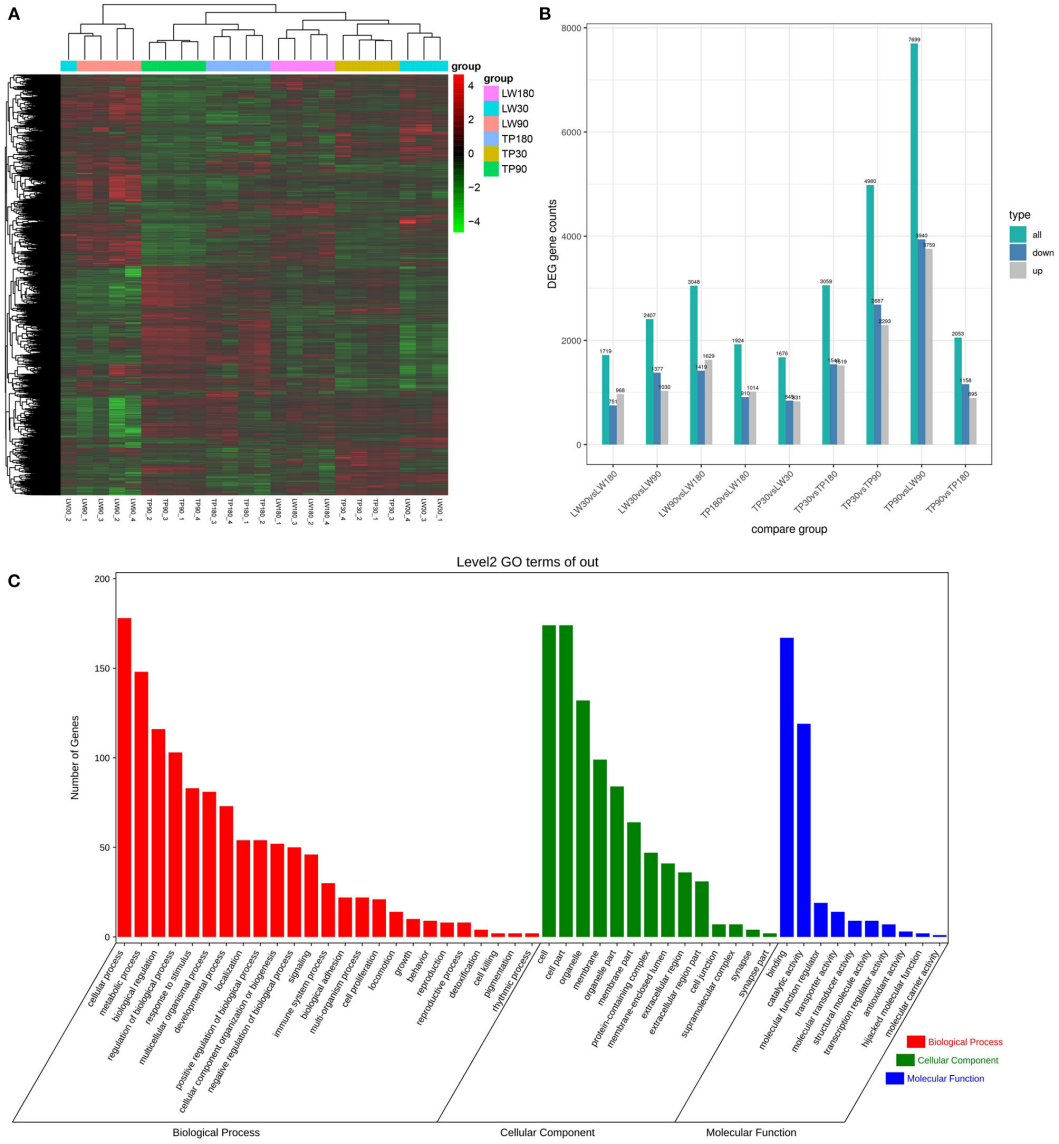
Analysis of differentially expressed genes (DEGs). **(A)** Plot of correlation coefficient among samples. **(B)** Histogram of the number of DEGS statistics for difference comparison combinations. **(C)** The column diagrams for gene ontology (GO) analysis of DEGs, the X-axis represents the functions of GO analysis and the Y-axis represents the numbers of DEGs.

The FPKM values of 24 RNA library samples were clustered using the mainstream hierarchical clustering method, and their rows were mean-scored (Z-score). Heat maps of gene correlations between the samples were analyzed and plotted. The results showed (Figure 1A) that the overall differences in expression of all genes were not prominent, and the correlation of gene expression between samples was good, reflecting the reliability of the experiments and sample selection, which can be applied to the subsequent differential gene screening and analysis.

### Analysis of Differentially Expressed Genes (DEGs)

**Figure 2:**
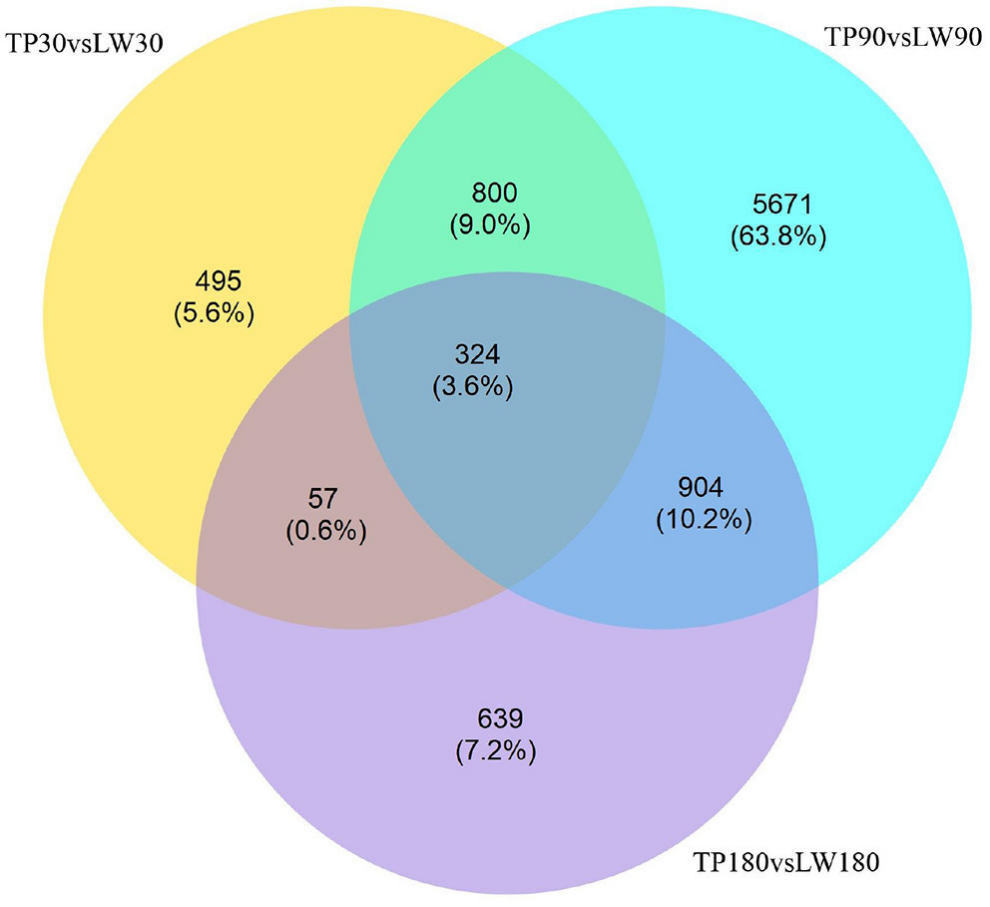
Differential genetic Venn diagram.

The conditions of Padj <0.01 and |log2FoldChange|>0 were used as screening criteria for differentially expressed genes, and the number of differential genes (including up-regulation and down-regulation) for each comparison combination was displayed in a histogram (Figure 1B), and a Venn diagram was used to show the overlap of differential genes between different comparison combinations to screen the differential genes of each comparison combination, and a total of 324 differential genes were screened in this experiment (Figure 2).

### Functional Analysis of DEGs

**Figure 3:**
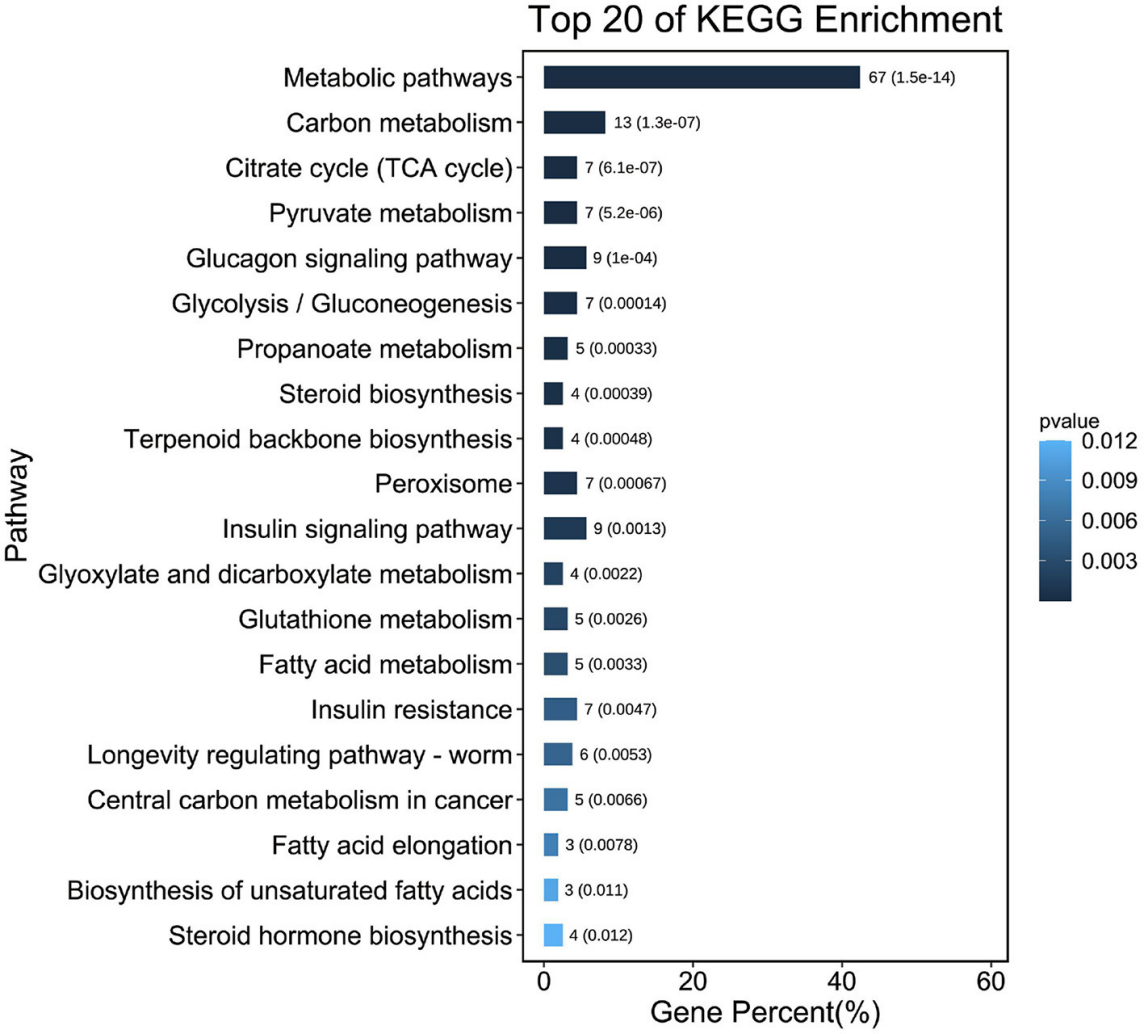
The column diagrams of top 20 Kyoto encyclopedia of genes and genomes (KEGG) analysis.

To further determine the functions of DEGS, all DEGS were functionally classified using GO annotations. The annotation results were divided into three parts: biological processes, cellular components, and molecular functions (Figure 1C). The top 5 of each section are shown as (1) Biological processes: (I) Cellular processes; (II) Metabolic processes; (III) Biological regulation; (IV) Regulation of biological processes; (V) Response to stimuli. (2) Cellular components: (I) cell; (II) cell part; (III) organelle; (IV) membrane; (V) organelle part. (3) Molecular functions: (I) binding; (II) catalytic activity; (III) molecular function regulator; (IV) transporter activity; (V) molecular transducer activity. KEGG analysis of DEG was also performed. As shown in Figure 3 and Supplementary Figure S1, KEGG was mainly enriched to metabolic pathways, carbon metabolism, TCA cycle, pyruvate metabolism, glucagon signaling pathway, and steroid biosynthesis and fatty acid metabolism pathways.

### RT-qPCR Validation of Transcriptome Data Results

**Figure 4:**
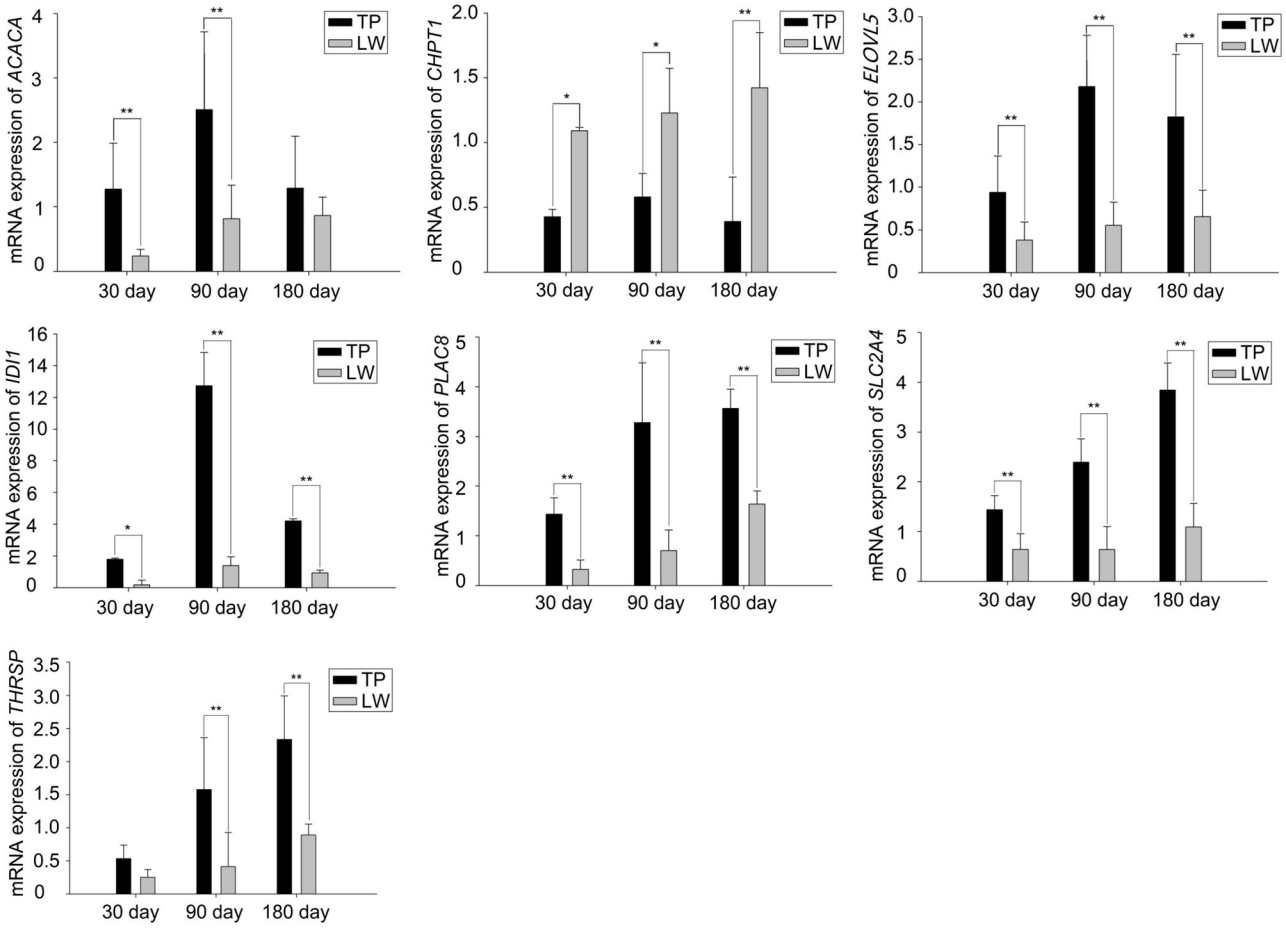
Real-time PCR validation of the DEGs analyzed by RNA-seq. The *symbol indicates the significant difference (*P* < 0.05). The **symbol indicates extremely significant difference (*P* < 0.01).

Results on further screening of 324 differential genes in the intersection of the TP30 vs. LW30 group, TP90 vs. LW90 and TP180 vs. LW180 groups, joint statistical analysis of FPKM values of differentially expressed genes related to lipid metabolism and fatty acid synthesis and comprehensive studies of their related functions, IDI1, PLAC8, ELOVL5, ACACA, SLC2A4, THRSP and CHPT1 were screened out. The RNA-seq data were consistent with those obtained by RT-qPCR (Figure 4). In addition, RT-qPCR analysis confirmed the DEG expression patterns observed in two pig breeds at different growth stages.

### Correlation Analysis of RT-qPCR Results With Backfat Thickness of Pigs

The results on backfat thickness measurement of Tibetan and York pigs (Table 1) showed that the backfat thickness of 180-day-old Tibetan carcasses was significantly higher than that of York pigs (*P* < 0.05).

**Table 1:** Comparison of backfat thickness determination.

**Species**	**Backfat thickness/mm**
Tibetan pig	21.27 ± 1.08a
York pigs	16.41 ± 1.16b

The correlation analysis between the backfat thickness data and the key candidate genes screened by transcriptome sequencing (Table 2) showed that the backfat thickness of York pigs was significantly positively correlated with IDI1, ACACA, ELOVL5, PLAC8, SLC2A4 and THRSP genes (*P* < 0.01), the correlation coefficients were 0.921, 0.935, 0.916, 0.934, 0.908, and 0.884, respectively. A highly significant negative correlation (*P* < 0.01) was found with CHPT1 gene, and the correlation coefficient was −0.928; the correlation analysis of each gene showed that CHPT1 gene showed a highly significant negative correlation with all genes, while all other genes showed a strong positive correlation with each other.

**Table 2:** Correlation analysis between backfat thickness and various genes in York pigs.

**Projects**	**Backfat thickness**	**IDI1**	**ACACA**	**ELOVL5**	**PLAC8**	**SLC2A4**	**THRSP**	**CHPT1**
Backfat thickness	1							
IDI1	0.921**	1						
ACACA	0.935**	0.959**	1					
ELOVL5	0.916**	0.991**	0.952**	1				
PLAC8	0.934**	0.959**	0.948**	0.967**	1			
SLC2A4	0.908**	0.928**	0.965**	0.910**	0.873*	1		
THRSP	0.884**	0.915**	0.967**	0.918**	0.915**	0.970**	1	
CHPT1	−0.928**	−0.889**	−0.885**	−0.890**	−0.955**	−0.855*	−0.898**	1

The *symbol indicates the significant difference (P <0.05). The **symbol indicates extremely significant difference (P <0.01).

Correlation analysis of Tibetan pig backfat thickness data with key candidate genes screened by transcriptome sequencing (Table 3) showed that the Tibetan pig backfat thickness was highly significant positive correlation with ACACA, SLC2A4 and THRSP genes (*P* < 0.01) with correlation coefficients of 0.868, 0.890 and 0.894, respectively, and negative correlation with CHPT1 gene, positive correlation with IDI1, ELOVL5, and PLAC8 genes, and the differences were not significant; CHPT1 gene was negatively correlated with all other genes, and all other genes showed positive correlations with each other.

**Table 3:** Correlation analysis between backfat thickness and various genes in Tibetan pigs.

**Projects**	**Backfat thickness**	**IDI1**	**ACACA**	**ELOVL5**	**PLAC8**	**SLC2A4**	**THRSP**	**CHPT1**
Backfat thickness	1							
IDI1	0.741	1						
ACACA	0.868**	0.953	1					
ELOVL5	0.823	0.700	0.930*	1				
PLAC8	0.766	0.999*	0.964	0.727	1			
SLC2A4	0.890**	0.706	0.991**	0.830	0.732	1		
THRSP	0.894**	0.934	0.991**	0.947*	0.947	0.976**	1	
CHPT1	−0.134	−0.743	−0.958*	−0.652	−0.768	−0.958*	−0.909*	1

The *symbol indicates the significant difference (P <0.05). The **symbol indicates extremely significant difference (P <0.01).

## Discussion

With the rapid development of high-throughput sequencing technology, transcriptome sequencing has been widely used in animal studies. Pooling and biological replicate are two conventional methods used in RNA-seq experiments. Pooling RNA-seq refers to mixing individuals in the same treatment group into one pool for sequencing, which is more efficient, less time-consuming, and incurs lower cost when there are many samples (16).

Various reports have shown that RNA-seq is also capable of detecting differentially expressed genes (DEGs) in mammals of different age and breeds (17, 18). Transcriptome comparison of adipose and muscle tissues from different pig breeds is a novel approach to study functional genes during adipogenesis (19). The adipose tissue is a complex and metabolically active tissue, and the adipocytes of which it is composed are a dynamic and highly regulated population of cells (20). Fat deposition is an important biological process in pig growth. Fatty traits are critical in pig production and are closely related to pork quality as well as the production efficiency and reproductive traits of pigs (21, 22).

Back fat thickness of pigs is an important indicator to determine the fat deposition in commercial pigs. Back fat thickness of pigs can directly reflect their body fat content (23). Investigation on measures to reduce back fat deposition is an important way to effectively promote genetic improvement of pigs. Therefore, it is important to explore the regulatory mechanisms and molecular mechanisms of pig back fat deposition.

At present, many studies have focused to identify mechanisms of fat deposition traits in local pig breeds at the molecular and genetic level but to date few studies have determined fat metabolism patterns in pigs at different growth stages. Therefore our study describes the scientific significance of studying of mechanisms involve in fat metabolism in pigs (24). In this study, we compared the dorsal fat tissues of Tibetan pigs (fatty breed) and York pigs (lean breed) at 30, 90 and 180 days of age by RNA-seq sequencing, and used Padj <0.01 and |log2FoldChange|>0 as the screening criteria for differentially expressed genes (DEGs), and obtained 800 DEGs by analysis in the 30 and 90 days of age groups. DEGs 904 were obtained in the 90-day-old group compared with the 180-day-old group; 57 DEGs were obtained in the 30-day-old group compared with the 180-day-old group; and 324 DEGs were obtained in the intersection of the three groups. In the process of fat deposition and metabolism, the specific expression of a large number of genes forms a complex regulatory network and signaling pathways. The functional annotation of differentially expressed genes revealed that the results of the remaining groups were basically consistent, except for the results of the 30-day-old and 180-day-old groups, which were mainly involved in monocarboxylic acid transport, lipid catabolism process, neutral lipid catabolism process, steroid metabolic process, carnitine shuttle and brown fat cell differentiation. The functional annotation of 57 DEGs obtained from the comparison of 30-day-old and 180-day-old groups was enriched to two GO entries of lipid transport and muscle organ development. Although the amount of data was small, the results fully indicated that lipid transport and muscle growth and development existed in the whole growth stage of pigs, reflecting the authenticity and accuracy of the data. The number of DEGs obtained by comparing the 30-day-old group with the 90-day-old group and the number of DEGs obtained by comparing the 90-day-old group with the 180-day-old group, i.e,57 <800 <904, because the process of lipid deposition in pigs is mainly reflected in the middle and late stages of growth, and this increment in number is fully consistent with the trend of growth and development of pigs, which further indicates the reliability of the data.

Seven key candidate genes were finally screened out based on the FPKM values of the differential genes integrated with their functional studies, among which acetyl-CoA carboxylase alpha (ACACA), an important key gene in the pathways of propionate metabolism, fatty acid biosynthesis, AMPK signaling pathway, insulin signaling pathway and glucagon signaling, plays an important role in the ab initio synthesis of fatty acids. The insulin signaling pathway not only plays an important role in muscle development and growth. It also plays an important role in the metabolism of carbohydrates and fats (25). It has been shown that in animal organisms, the ACACA gene is highly expressed in tissues with strong fatty acid metabolic activity, and the acetyl coenzyme A carboxylase (ACACA) gene plays an important role in lipid metabolism by promoting fatty acid biosynthesis (26). The results of our study also showed extremely high expression in porcine dorsal lipid tissues, which is consistent with the findings of previous studies (27, 28). Solute carrier family 2 member 4 (SLC2A4), also known as glucose transporter protein 4 (GLUT4), is an important transporter protein for cellular uptake of glucose, mainly expressed in muscle and adipose tissues, involved in active cellular uptake of glucose across membranes, and plays an important role in glucose metabolism, and was enriched in the present assay in AMPK signaling pathway, insulin signaling pathway and adipocyte factor signaling pathways. KEGG results showed the meaningful signaling pathways were mainly focused on glucose metabolism. Some of these pathways, such as ‘glycolysis/gluconeogenesis', although they are not directly related to lipid synthesis and metabolism, glucose and lipids can be interconverted and jointly participate in the tricarboxylic acid cycle. Therefore, glucose metabolic processes may have an indirect effect on lipid metabolism. The KEGG results showed the pathways were mostly concentrated on glucose metabolism. some of these pathways, such as ‘Glycolysis/Gluconeogenesis', although they are not directly related to lipid synthesis and metabolism, glucose and lipids can change Therefore, the process of glucose metabolism can have an indirect effect on lipid metabolism (23). Thyroid hormone response protein (THRSP), alias LPGP1, SPOT14, THRP, is induced by thyroid hormone expression and is closely related to lipid deposition in animals. The changes in THRSP gene mRNA content in tissues directly affect the ability of tissues to synthesize lipids. In previous studies, THRSP was considered as a candidate gene for growth traits and played an important role in adipogenesis, and was significantly expressed in subcutaneous adipose tissue of yaks. The sequencing results in this paper showed that THRSP was also highly expressed in pig dorsal lipid tissue, and the conclusion was consistent with it, which is of scientific significance for further study as a key candidate gene for fat deposition.

The choline phosphotransferase 1 gene (CHPT1), alias CPT and CPT1, was first successfully cloned in humans in 2000, but its cloning in pigs has not yet been reported. The CHPT1 gene is involved in lipid metabolism, transferring part of the phosphorylcholine from CDP-Choline to lipid The CHPT1 gene is involved in the process of lipid metabolism and can transfer part of the phosphorylcholine from CDP-Choline to the raw material of lipid production (diglycerides) to produce phosphatidylcholine, which has the biological functions of regulating the normal synthesis and metabolism of lipids and reducing the concentration of cholesterol and triglyceride levels (29); the CHPT1 gene expression differs more than 20-fold in the dorsal fat of Rongchang pigs at 240 days of age and 30 days of age (30). In the present study, the CHPT1 gene was differentially expressed in dorsal fat tissues at different age stages and was negatively correlated with pig backfat thickness. It is speculated that this gene may be a potential gene for fat deposition traits in pigs.

The mRNA expressions of IDI1, PLAC8 and ELOVL5 genes in this study, although is not significantly different in the correlation analysis with backfat thickness in Tibetan pigs, was still positively correlated, suggesting that these three genes are potential candidates for regulating fat deposition traits in pigs. Isopentenyl-diphosphate isomerase 1 (isopentenyl-diphosphate delta-isomerase 1, IDI1), was screened in GO entries regulating the terpene skeleton biosynthesis pathway and cholesterol biosynthesis process. It is a cytoplasmic enzyme involved in the biosynthesis of isoprenoids, including cholesterol, and is associated with fatty acid metabolism, lipoprotein signaling and cholesterol metabolism (31). The expression of placenta-specific protein 8 (PLAC8) has not been shown in pigs, but in this experiment the expression of PLAC8 gene on dorsal lipid tissues showed an increasing trend with increasing age.PLAC8 affects the differentiation and formation of brown adipocytes and is an important regulator in the process of adipogenesis (32). The elongase of very long-chain fatty acids 5 (ELOVL5), as the initiator and rate-limiting enzyme of fatty acid synthesis, plays an important role in the synthesis of fatty acids in animals and is involved in the extension of fatty acid chains and regulation of fatty acid synthesis, respectively, affecting the body's blood glucose and lipid concentrations, and is extremely relevant to fat deposition traits. It is extremely relevant to fat deposition traits and is important to be further validated as a key gene for fat deposition traits.

## Conclusions

In this study, Tibetan and York pigs at different growth stages were used as test subjects, and their dorsal fat tissues were collected for transcriptome sequencing, and a total of 32,747 differentially expressed genes were screened. The range was narrowed down to 324 differentially expressed genes by screening the differentially expressed genes between breeds and days of age. The functional enrichment analysis revealed significant enrichment to the entries of metabolic pathways, steroid biology, unsaturated fatty acid synthesis pathway and lipid metabolism. And finally, seven differential genes were screened for joint validation of the regulatory role of pig backfat thickness on fat deposition, and it was hypothesized that Tibetan pig backfat thickness was highly significantly and positively associated with ACACA, SLC2A4 and THRSP genes, and York pigs were highly and positively associated with IDI1, ACACA, ELOVL5, PLAC8, SLC2A4 and THRSP genes, and Tibetan pigs Both Tibetan pigs and York pigs showed highly significant negative correlations with CHPT1 gene. This study initially revealed the differentially expressed genes and signaling pathways affecting fat deposition and metabolism in Tibetan and York pigs, and laid a theoretical foundation for the improvement of fat deposition ability in pigs.

## Data Availability Statement

The datasets presented in this study can be found in online repositories. The names of the repository and accession number can be found below: SRA database, accession number SRP372504.

## Ethics Statement

The animal study was reviewed and approved by the Animal Welfare Committee of Tibet Agriculture and Animal Husbandry University.

## Author Contributions

PS and ZW conceived the experiments. PS and YC performed the experiments and analyzed the data. XG and MZ wrote and revised the paper. JZ and YY revised the paper. MD reviewed the paper. All authors have read and agreed to the published version of the manuscript.

## Funding

This study was supported by the National Natural Science Foundation of China (32160773), Major Science and Technology Projects of the Tibet Autonomous Region (XZ202101ZD0005N), the Graduate Innovation Program of Tibet Institute of Agriculture and Animal Husbandry (YJS2022-21), and Key R&D Plan of Bayi District, Nyingchi City (2021-GX-SY-01).

## Conflict of Interest

The authors declare that the research was conducted in the absence of any commercial or financial relationships that could be construed as a potential conflict of interest.

## Publisher's Note

All claims expressed in this article are solely those of the authors and do not necessarily represent those of their affiliated organizations, or those of the publisher, the editors and the reviewers. Any product that may be evaluated in this article, or claim that may be made by its manufacturer, is not guaranteed or endorsed by the publisher.
