# Supplementary figures and images for: Transcriptomics-Based Study of Differentially Expressed Genes Related to Fat Deposition in Tibetan and Yorkshire Pigs
Source: Front Vet Sci. 2022 Jun 9;9:919904. doi: 10.3389/fvets.2022.919904 (PMC9218471; doi:10.3389/fvets.2022.919904)

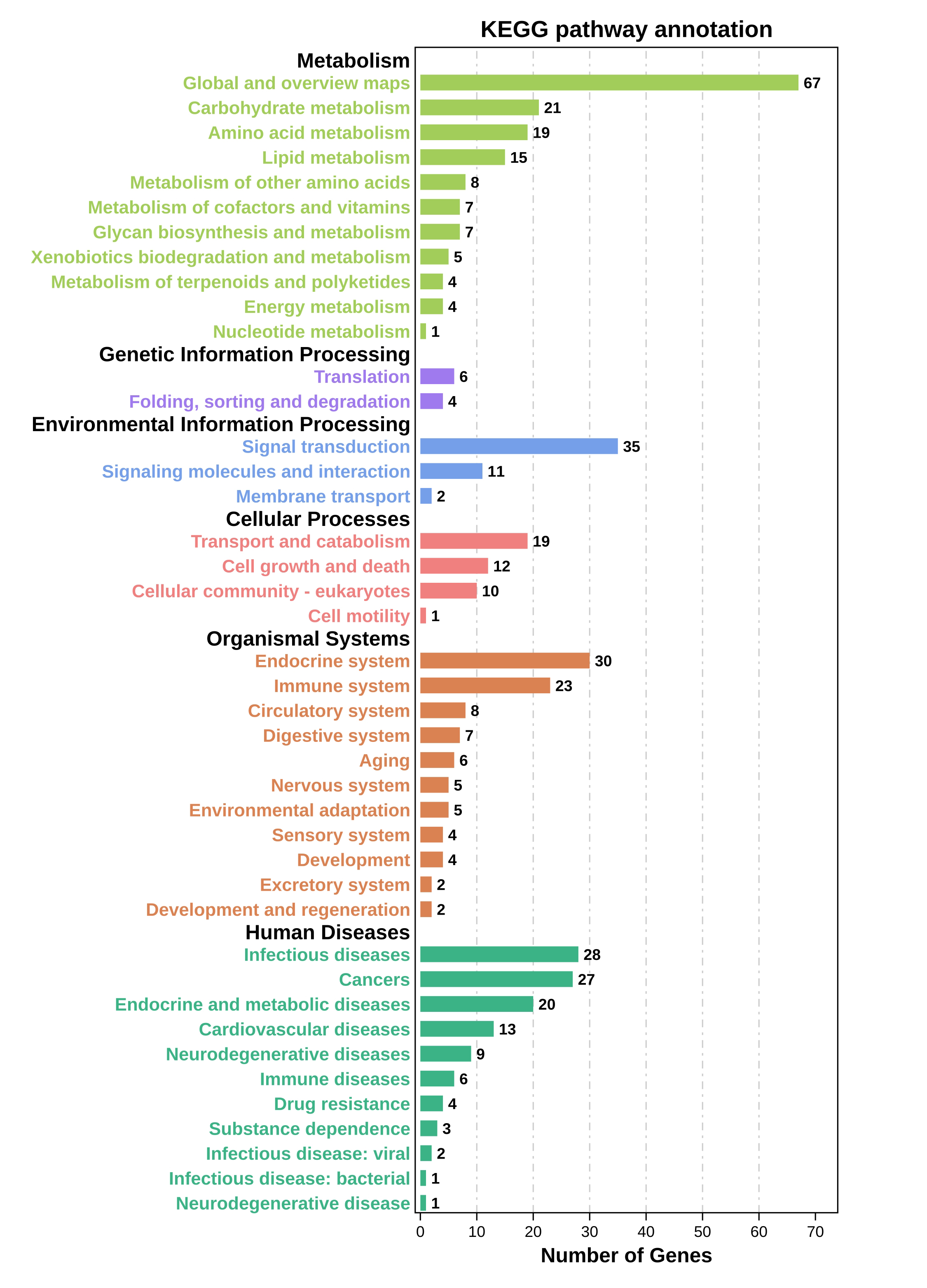

Supplement: Supplementary file 1 [file Image_1.JPEG]
